# Sociodemographic and clinical profiles of patients receiving home care and the occurrence and management of healthcare-associated infections: a cross-sectional study

**DOI:** 10.1590/1516-3180.2024.0156.03072024

**Published:** 2025-02-07

**Authors:** Joelma Lacerda de Sousa, Antonio Rosa de Sousa, Jaqueline Carvalho e Silva Sales, Rosilane de Lima Brito Magalhães, Denise de Andrade, Andréia Rodrigues Moura da Costa Valle

**Affiliations:** I Postgraduate Nursing Program, Universidade Federal do Piauí (UFPI), Teresina (PI), Brazil.; II Master’s Student, Postgraduate Nursing Program, Universidade Federal do Piauí (UFPI), Teresina (PI), Brazil.; III Professor, Postgraduate Program in Family Health, Universidade Federal do Piauí (UFPI), Teresina (PI), Brazil.; IV Professor, Postgraduate Nursing Program, Universidade Federal do Piauí (UFPI), Teresina (PI), Brazil.; V Professor, Ribeirão Preto College of Nursing, Universidade de São Paulo (USP), Ribeirão Preto (SP), Brazil.; VI Professor, Postgraduate Nursing Program, Universidade Federal do Piauí (UFPI), Teresina (PI), Brazil.

**Keywords:** Homebound persons, Home care services, Infection control, Cross infection, Cross-sectional studies, Health profile, Home care, Occurrence of infections, Health Care-Associated Infections, Retrospective study

## Abstract

**BACKGROUND:**

Home care is increasingly adopted worldwide to improve patients’ quality of life and reduce the burden on hospitals. However, the risk of healthcare-related infections in home settings is a growing concern that necessitates further investigation and preventive measures.

**OBJECTIVES:**

We aimed to describe the sociodemographic and clinical profiles of home care patients, determine the incidence and management of healthcare-associated infections at home, and evaluate the risk factors.

**DESIGN AND SETTING:**

This quantitative, observational, analytical, cross-sectional study was conducted in Teresina, PI, Brazil.

**METHODS:**

Data were collected from 130 patients receiving home care between April 2016 and September 2020 in the state capital of Northeast Brazil. The data were retrospectively collected from hospital records using a previously validated form and analyzed.

**RESULTS:**

The cohort predominantly comprised men (53.1%), older adults (53.1%), and patients with neurological disorders (61.9%). Healthcare-associated infections were prevalent in 46.2% of home care patients, with respiratory infections being the most common (47.2%). Clinical diagnoses were made in 66.7% of these patients. Patients with female caregivers, with a tracheostomy, using invasive feeding devices for >6 months, and with a greater degree of dependence were more predisposed to infections. Adult patients, those with young adult caregivers, those who received long-term home care, and those who required prolonged tracheostomy were also at increased risk.

**CONCLUSION:**

This study underscores the home care patient profiles, prevalence of associated infections, and risk factors. Preventive measures and specific interventions are needed to enhance home care quality and reduce the infection risk.

## INTRODUCTION

Home care (HC) practices, especially those for patients with acute and chronic illnesses, palliative care needs, and functional disabilities, are expanding globally. Successful programs that offer health services directly to patients’ homes demonstrate this trend.^1-2^ These initiatives aim to transition care from hospital settings, often viewed as challenging, to the home environment, aiming to improve quality of life and facilitate recovery.^3^

The benefits of home hospitalization include reduced hospital discharges, decreased hospital congestion, lower costs, increased turnover of hospital beds, and complementary care.^4^ Furthermore, HC enhances the humanization of care and improves the quality of life, by involving family members in patient care and strengthening the relationships between the healthcare team, the patient, and their families.^5^

Evaluating the effectiveness of HC services, particularly concerning healthcare-associated infections (HAIs), is essential.^6^ HAIs are the most frequent adverse outcomes in healthcare delivery and pose a serious public health problem owing to their high morbidity and mortality and significant financial burden on healthcare systems. Cross-contamination, the primary transmission route of microbial infections, is a common issue that can also occur during HC, underscoring the need for thorough investigation and prevention of these infections in all healthcare settings.^7^

National and international ordinances regulate the surveillance and monitoring of infections in hospital settings, establishing specific standards and guidelines to ensure patient safety and prevent the spread of disease. These ordinances establish hygiene protocols, cleaning and disinfection procedures, and provide guidelines for the appropriate use of personal protective equipment by healthcare professionals.^8^ Extensive scientific literature exists on protocols for preventing and controlling hospital infections. However, investments in HC environments are limited and based on fragmented information, leading to the lack of robust prevention, control, and standardized monitoring programs for infections.^9^

In Brazil, HC is part of the “Better at Home” program in the Urgency and Emergency Network, regulated by Ordinance No. 825/2016 of the Ministry of Health (MH). The ordinance established the Home Care Service (HCS) to manage and operationalize multidisciplinary home care teams (MHCTs). The service is organized into three modalities: HC 1, focusing on primary care, and HC 2 and HC 3, which cater to medium- and high-complexity patients requiring assistance at least once a week.^10^

Despite this, Brazilian scientific research on HC primarily focuses on the characterization of the service, with limited attention given to infections in the home environment.^11^ The national program for preventing and controlling healthcare-related infections only briefly mentions HC, challenging health professionals to implement HAI prevention and control measures at home due to the lack of specific theoretical guidelines.^12^

Considering the complexity of the home environment, understanding these patients and the occurrence and management of HAIs at home is essential, given the risk factors similar to those in the hospital settings and the specific factors that require further studies.^7^

## OBJECTIVE

This study aimed to describe the sociodemographic and clinical profiles of patients receiving home care, investigate the occurrence and management of HAIs at home, and evaluate the factors associated with these infections.

## METHODS

### Type of study

The study employs a quantitative, observational, analytical, and cross-sectional design, characterized by the systematic analysis of numerical data and detailed observations captured at a specific time point.^13^ This study adhered to the Strengthening the Reporting of Observational Studies script in Epidemiology guidelines, to improve the quality and transparency of reports on epidemiological observational studies.^14^

### Study location

The study was conducted in the state capital of Northeast Brazil. Data were collected from the medical records of emergency hospitals. This hospital, managed by the Municipal Health Foundation in collaboration with the State Health Department, exclusively provides care through the Unified Health System (UHS).

This hospital was selected as at the time of data collection, it was the only hospital accredited by the HCS and operated two MHCTs. Patient records are maintained in printed form by the hospital’s medical and statistical archive service, and the MHCTs discharge patients from the same health unit.

### Population, sample, and sampling

The study population included all medium- and high-complexity patients who received MHCT care from April 2016 to September 2020. This period was established in line with the implementation of Ordinance No. 825/2016 of the MS, which redefined HC in the context of the UHS and updated the qualified teams despite the program having existed since 2011.^10^ Data collection concluded after reviewing all available medical records within this pre-determined timeframe, resulting in a total of 130 patients.

### Inclusion and exclusion criteria

The study included patients of both sexes and all ages registered at the HC, treated by the MHCT, and discharged from the hospital. Meanwhile, patients with illegible data or inconsistent medical record filling, owing to the difficulty in extracting information for analysis, were excluded.

We selected patients who exhibited signs and symptoms of infection only after 72 hours of hospital discharge to distinguish HAIs at the hospital from HAIs at home.^15^ Additionally, we verified whether patients had been hospitalized for infection before receiving assistance from the MHCTs. This step aimed to differentiate between HAIs at the hospital, HAIs at home, and community-acquired infections, thus reducing the bias in attributing infections from other sources, such as HAIs acquired at home.

### Data collection technique

Data were collected retrospectively from the medical records of a local hospital using a standardized form. Information was gathered from identification forms, admission and discharge summaries, progress notes recorded by healthcare professionals during care, and test results attached to the medical records. Data were collected from August 10 to September 22, 2020, with the participation of three researchers: a master’s student in nursing and two previously trained nursing undergraduates. The researchers collaborated as a team during the collection process, often working in pairs or trios, after establishing a prior alignment during the pilot testing phase.

The form used in the study was validated by experts and was utilized in the dissertation titled, “Occurrence of infections among patients in home care: insights for epidemiological surveillance.” This instrument comprises four distinct sections: sociodemographic data (8 fields), housing data (2 fields), clinical data (28 fields), and infection data (17 fields), totaling 55 fields.^16^

### Variables

The independent variables were classified into three distinct groups: sociodemographic, clinical, and infection related. The sociodemographic group included variables such as sex, age group (years), education (years), marital status, caregiver’s sex, caregiver’s age group, relationship with the caregiver, and area of residence. In the clinical group, we included variables such as primary pathology, length of hospital stay (in days), duration of home care (in days), use of venous catheters, duration of tracheostomy (in days), feeding route, duration of invasive feeding device use, route of elimination, use of an indwelling urinary catheter, degree of dependence (assessed using the Spanish scale), presence and number of pressure injuries, and current comorbidities. In the infection-related group, variables included the occurrence of infection, number of infections, topography of infections, method of infection diagnosis, infection management, results of culture examinations, antibiograms, antimicrobial multi-resistance, and infection recurrence.

We also considered the occurrence of infection as a dependent variable after performing inferential statistical analyses to establish significant associations between several variables and HAI occurrence at home.

### Data processing and analysis

Initially, we manually organized the forms, tabulated the data, and performed double entry using Microsoft Excel^®^ version 2016 (Microsoft Corporation^®^, Redmond, WA, USA). Subsequently, we imported the data into the Statistical Package for the Social Sciences software version 22.0 for Windows (IBM^®^, Armonk, NY, USA) for further analysis.

For statistical analysis, we conducted univariate analyses of independent variables, including frequency distribution (mean, standard deviation, and median). Fisher’s exact test was used to investigate the association between sociodemographic and clinical data (categorical variables) and the occurrence of HAIs at home. Furthermore, we employed Poisson logistic regression to calculate the adjusted odds ratio (AOR) for variables with a P value of less than 0.05. Subsequently, we used the Mann-Whitney U test to compare sociodemographic and clinical data (continuous variables) with the occurrence of HAIs at home.

### Ethical and legal aspects

This study was approved by the Research Ethics Committee of the Univerisdade Federal do Piauí under opinion number 3,982,462, on April 21th, 2020.

## RESULTS

After excluding 45 medical records due to incomplete data, the final study sample comprised 130 patients from the initial 175 recruited at the institution providing HC services.

The results indicated a majority of male patients (53.1%) and a predominance of older adults, with over half of the sample (53.1%) being aged ≥60 years. Most patients had 1 and 9 years of education (47.2%) and were single or had no spouse (61.1%). The caregivers were predominantly women (83.2%) and primarily adults (74.7%), with the most common relationship being with the caregiver of a son or daughter (31%). Urban residences were the predominant location compared with rural residences (92.3%) (Table 1).

**Table 1:** Demographic characterization of home care patients

Variables	n (%)	M ± SD	MD
**Sex**			
Male	69 (53.1)		
Female	61 (46.9)		
**Age group (in years)**			
Up to 19 years (youth)	7 (5.4)	59.42 ± 23.49	61
20–59 years (adults)	54 (41.5)		
≥ 60 years (older adult)	69 (53.1)		
Education (in years)*			
Illiterate	33 (37.1)		
1–9 years of education	42 (47.2)		
> 9 years of education	14 (15.7)		
Marital status*			
Single, widowed, or separated	69 (61.1)		
Married or in a stable union	44 (38.9)		
Caregiver’s gender*			
Male	21 (16.8)		
Female	104 (83.2)		
**Caregiver’s age group**			
Up to 19 years (youth)	2 (1.5)	42.6 ± 14.48	41.5
20 to 59 years (adults)	97 (74.7)		
≥ 60 years (older adult)	31 (23.8)		
Relationship with caregiver*			
Father	7 (5.9)		
Mother	22 (18.5)		
Son or daughter	37 (31.1)		
Brother or sister	15 (12.6)		
Spouse	14 (11.8)		
Other	24 (20.1)		
Residential area*			
Urban	119 (92.3)		
Rural	10 (7.7)		

M = mean; SD = standard deviation; MD = median;

*Quantity of data not available: education (in years) (n = 41), marital status (n = 17), sex (caregiver) (n = 5), relationship with caregiver (n = 11), and residential area (n = 1).

Of the 130 patients assessed, 61.9% had a neurological pathology. The average hospital stay was 59.78 days, while the average home care duration was 258.40 days. Most patients did not use venous catheters (95.3%) or required respiratory support (83.8%). Approximately 52.3% of patients used a tracheostomy (average duration: 360.17 days), while 53.1% used a nasoenteral tube for feeding, typically for up to 6 months (66.7%). Physiological elimination was predominant (95.3%), and the majority were classified as totally dependent (85.2%) based on the Spanish Dependence Scale scores. Pressure injuries were observed in 76.9% of the patients, with the majority having only one injury (58.3%). Approximately one-third of the patients had comorbidities (35.2%). HC outcomes included discharge with improvement (43.1%) and death (33.6%) (Table 2).

**Table 2:** Clinical characteristics of home care patients

Variables	n (%)	M ± SD	Md
Primary pathology*			
Neurological pathologies	78 (61.9)		
Respiratory pathologies	14 (11.1)		
Infectious pathologies	7 (5.6)		
Other pathologies	27 (21.4)		
Length of hospital stay (in days)*		59.78 ± 63.18	43.00
Length of home care (in days)*		258.40 ± 309.46	131.50
Use of venous catheters*			
Yes	6 (4.7)		
No	123 (95.3)		
**Breathing**			
Unassisted	109 (83.8)		
Assisted	21 (16.2)		
**Use of tracheostomy**			
Yes	68 (52.3)		
No	62 (47.7)		
Duration of tracheostomy use (in days)*		360.17 ± 403.17	192.50
**Feeding route**			
Oral	33 (25.4)		
Nasoenteral tube	69 (53.1)		
Gastrostomy	28 (21.5)		
Duration of invasive feeding device use *			
Up to 6 months	48 (66.7)		
More than 6 months	24 (33.3)		
Elimination route*			
Physiological	122 (95.3)		
Colostomy	4 (3.1)		
Cystostomy	2 (1.6)		
**Use of indwelling urinary catheter**			
Yes	42 (32,3)		
No	88 (67.7)		
Degree of dependency (Spanish scale)*			
Independent	4 (3.1)		
Partially dependent	15 (11.7)		
Totally dependent	109 (85.2)		
**Presence of pressure ulcer**			
Yes	100 (76.9)		
No	30 (23.1)		
Number of pressure ulcers*			
One	56 (58.3)		
Two	18 (18.8)		
Three or more	22 (22.9)		
Current comorbidities*			
Yes	43 (35.2)		
No	79 (64.8)		
**Discharge with improvement**			
Hospital readmission	20 (17.2)		
Death	39 (33.6)		
Discharge with improvement	50 (43.1)		
Others	7 (6.0)		

M = mean; SD = standard deviation; Md = median;

*Quantities of data unavailable: primary pathology (n = 4), length of hospital stay (in days) (n = 28), length of home care (in days) (n = 10), duration of tracheostomy use (in days) (n = 14), duration of invasive feeding device use (n = 25), elimination route (n = 2), degree of dependency (Spanish scale) (n = 2), number of pressure ulcers (n = 4), and current comorbidities (n = 8).

Approximately 46.2% of the patients developed HAIs at home, with the majority (75.0%) being affected by only one type of infection, primarily a respiratory infection (47.2%). Approximately 66.7% of the patients were clinically diagnosed with HAI, but only 47.3% received antibiotic therapy, while culture and antibiogram testing were performed in 11.7% of the patients. All seven identified microorganisms exhibited multidrug resistance (100%). Twenty-two patients (36.7%) developed infection recurrence, while 38 patients (63.3%) did not experience recurrence (Table 3).

**Table 3:** Characteristics of infections and patient management in home care

Variables	n (%)
**Occurrence of infection**	
Yes	60 (46.2)
Not	70 (53.8)
**Number of infections**	
Two types of infections	45 (75.0)
Method of infection diagnosis	15 (25.0)
Infection site*	
Urinary tract infection	24 (32.4)
Respiratory infection	35 (47.2)
Skin infection	6 (8.1)
Surgical site infection	1 (1.4)
Bloodstream infection	2 (2.7)
Gastrointestinal infection	5 (6.8)
Others	1 (1.4)
**Method of infection diagnosis**	
Clinical examination	40 (66.7)
Laboratory test	9 (15.0)
Clinical examination and laboratory test	11 (18.3)
**Management of infection†**	
Hospital readmission	13 (23.6)
Antibiotic therapy	26 (47.3)
More than one management	16 (29.1)
**Performance of culture tests**	
Yes	7 (11.7)
Not	53 (83.3)
**Performance of antibiograms**	
Yes	7 (11.7)
Not	53 (83.3)
**Multidrug resistance**	
Yes	7 (100)
Not	-
**Infection recurrence**	
Yes	22 (36.7)
Not	38 (63.3)

*In the “infection site” variable, patients who presented more than one type of infection were considered; †Quantity of data not available: management of infection (n = 5).

Significant associations were found between several variables and HAIs at home, with Fisher’s exact test indicating a predisposition in patients assisted by female caregivers (P = 0.001), who required tracheostomies (P = 0.048), with prolonged use of invasive feeding devices (P < 0.001), and had a higher degree of dependence (P = 0.018) **(Table 4**).

**Table 4:** Association between sociodemographic and clinical variables with the occurrence of HAIs at home

Occurrence of infection related to home care			
Variables	Yes n (%)	Not n (%)	P value*
Caregiver’s Sex**			
Male	3 (2.4)	18 (14.4)	**0.001**
Female	56 (44.8)	48 (38.4)	
**Use of tracheostomy**			
Yes	37 (28.5)	31 (23.8)	**0.048**
No	23 (17.7)	39 (30.0)	
Duration of invasive feeding device usage **			
Up to 6 months	16 (22.2)	32 (44.4)	**< 0.001**
More than 6 months	20 (27.8)	4 (5.6)	
Degree of dependency (Spanish scale)**			
Independent or partially dependent	4 (3.1)	15 (11.7)	**0.018**
Totally dependent	55 (43.0)	54 (42.2)	

*Fisher’s exact test;

**Quantities of data unavailable: caregiver’s sex (n = 5), duration of invasive feeding device usage (n = 25), and degree of dependency (Spanish scale) (n = 2).

Poisson logistic regression analysis revealed that patients assisted by female caregivers were approximately seven times more likely to acquire the infection (AOR = 6.611). Tracheostomy use increased the likelihood of infection by more than four times (AOR = 4.335). Patients who used invasive feeding devices for more than 6 months had a 15-fold increased risk of infection (AOR = 15.044) than those who used such devices for a shorter duration. Patients with total dependence had approximately four times higher odds of infection (AOR = 3.892) than those who were independent or partially dependent (Table 5).

**Table 5:** Logistic regression for the occurrence of HAIs at home

Patient with healthcare-associated infection at home			
Variables	AOR	95% CI	P value*
Caregiver’s sex**			
Male	-	-	**0.004**
Female	6.611	1.826–23.933	
**Use of tracheostomy**			
Yes	4.335	1.045–17.977	**0.043**
No	-	-	
Duration of invasive feeding device usage **			
Up to 6 months	-	-	**0.002**
More than 6 months	15.044	2.759–82.033	
Degree of dependency (Spanish scale)**			
Independent or partially dependent	-	-	**0.024**
Totally dependent	3.819	1.191–12.246	

AOR = adjusted odds ratio; 95%CI = 95% confidence interval;

*Poisson logistic regression;

**Quantities of unavailable data: caregiver sex (n = 5), duration of invasive feeding device usage (n = 25), and degree of dependency (Spanish scale) (n = 2).

The comparative analysis further revealed that adult patients (54.57 ± 24.91), those cared for by young adult caregivers (38.76 ± 13.43), those who received prolonged home care (386.21 ± 362.64), and those who required prolonged tracheostomy (470.34 ± 459.42) were more susceptible to infections (Table 6).

**Table 6:** Comparison of sociodemographic and clinical variables with occurrence of HAI at home

Occurrence of infection related to healthcare at home			
Variables	Yes	Not	P value*

	M ± SD*	M ± SD	
Age	54.57 ± 24.91	63.61 ± 21.58	**0.049**
Caregiver’s age	38.76 ± 13.43	44.90 ± 14.97	**0.014**
Length of home care (in days)**	386.21 ± 362.64	146.00 ± 198.21	**< 0.001**
Duration of tracheostomy use (in days)**	470.34 ± 459.42	219.65 ± 265.85	**0.001**

M = mean; SD = standard deviation;

*Mann-Whitney U test;

** quantities of data unavailable: length of home care (in days) (n = 10) and duration of tracheostomy use (in days) (n = 14).

## DISCUSSION

Some similarities with previous studies were observed in the sociodemographic characteristics of home-cared patients, revealing a predominance of older adults and adults in the sample.^17^ Furthermore, the literature shows that the rate of home admissions increases proportionally with age.^12^

In the context of relationships with caregivers, a predominance of women as primary caregivers emerged, especially as they approached the later stages of adulthood and the reproductive phase. These findings, commonly reported in the scientific literature, indicate that despite advancements in gender equality, women remain predominantly responsible for caring for sick family members.^18^

With regard to the main pathology, a high incidence of neurological disorders, primarily caused by cerebrovascular accidents (CVAs), has emerged. These disorders are associated with several risk factors, such as high blood pressure, diabetes, smoking, high cholesterol, obesity, sedentary lifestyle, family history, and advanced age. These findings underscore the importance of adopting healthy habits, preventing chronic diseases, and continuously monitoring patients to improve their health behaviors and prevent CVA recurrence.^19^

The relevance of home care was evident as patients stayed longer at home than in the hospital. This suggests that home-based treatments are more suitable for these patients. According to the literature, home care is not only beneficial to patients, but is also economically advantageous for the healthcare system, thereby supporting the need for its expansion and enhancement.^20^

Just over half of the treated patients underwent tracheostomy, required invasive feeding devices, and had a high degree of dependence. This finding highlights the complexity of public-serving HC. These data corroborate those of a study in Brazil that demonstrated a high volume of outpatient home procedures, including consultations, multidisciplinary care, nursing care, and physiotherapy.^11^

One concerning issue was the occurrence of pressure injuries in home care patients. This can cause pain and distress in patients and increase the burden on caregivers. Literature suggests that adherence to pressure-relief schedules may be lower in home settings due to caregiver unawareness of pressure injuries and the importance of repositioning. These findings emphasize the need for new approaches to prevent such injuries.^21^

The primary outcome that emerged was discharge with improvement, highlighting the importance of HC. HC is fundamental owing to its unique approach in delivering care, which requires a thorough examination of the diverse experiences of both users and caregivers. This perspective goes beyond the simple organization of the network and its pre-established flows, demanding strategies capable of recognizing and addressing the needs of users and their families.^22^

The incidence rate of HAIs at home was high. However, national and international studies on the occurrence of these infections in home care patients are limited, outdated, and present different methodologies, making it difficult to generalize the results. However, these infections should be explored more thoroughly in the literature to facilitate their integration in both care practices and educational contexts.^23^

With regard to the topography and recurrence of infections, the present study revealed a predominance of respiratory infections, with a recurrence rate of more than one-third. These findings align with those of a study involving 199,462 patients from 8,255 home healthcare agencies, which identified that infections were responsible for 17% of unplanned hospitalizations. Respiratory infections, wound infections or deterioration, and urinary tract infections are the leading causes, and three of the six main reasons for unplanned hospitalization are related to infections.^24^

Scientific literature provides strategies for combating these infections. Effective approaches include employing a trained infection prevention professional who implements strict surveillance criteria and utilizes different methodologies to identify and combat the underreporting of these infections. Establishing an infection prevention and epidemiology committee to develop annual prevention plans and adhering to appropriate surveillance definitions can aid in identifying at-risk populations and providing accurate data using evidence-based surveillance.^25^

In managing HAIs at home, clinical diagnosis and antibiotic therapy were predominant, with limited use of culture and antibiogram tests. Although clinical diagnosis is useful, complementing it with cultures and antibiograms is crucial for guiding precise antibiotic treatment. Without adequate microbiological investigation, the indiscriminate prescription of antibiotics can generate multidrug-resistant strains, underscoring the need to reduce inappropriate antibiotic use through effective diagnosis and responsible management.^26^

Other statistical analyses did not find an association between female caregivers and a greater risk of infection in home care patients, underscoring the need to consider these data with caution. These findings may be attributed to the workload associated not only with caring for patients in a home care setting but also with addressing the individual needs of children, husbands, and other dependents, which can impact the quality of care provided by the female caregiver.^18,27^

The influence of patient and caregiver age on the occurrence of infections during comparative analysis may be explained by several factors. First, younger patients might be less susceptible to infections than older patients, potentially leading to neglect in care.^28^ The age of caregivers and the occurrence of infections, especially in those cared for by younger individuals, can be attributed to the frequent demands of daily responsibilities. These responsibilities can affect the quality of care provided and even the caregiver’s health.^29^

The association between total dependence and infections corroborates the above findings, as these patients may require more intensive and continuous care, which can overwhelm caregivers, particularly those with additional daily responsibilities. Furthermore, the literature that highly dependent individuals are more susceptible to health complications and require constant assistance with daily living activities, increasing their exposure to the risk factors for infections and further compromising their health.^30^

The relationship between the occurrence of infections in tracheostomized patients can be attributed to their increased susceptibility to infections due to the direct airway access, which facilitates pathogen entry to the body.^31^ Conversely, the incidence of infections associated with tracheostomy use can be attributed to the higher likelihood of microorganism colonization on these devices over time, which can consequently increase the risk of infectious complications and compromise patient health.^32^

The use of invasive feeding devices for more than 6 months increases the risk of infection. This finding may be related to the patient’s greater exposure to pathogenic agents, colonization of the device by microorganisms over time, and difficulty in maintaining hygiene. Difficulty in maintaining hygiene can cause skin irritation or tissue damage and encourage the entry of infections.^33^

Notably, the difficulty in maintaining hygiene, which may also have influenced the occurrence of infections in patients who spent more time in home care, may have been aggravated by the lack of adequate structures in the home environment. Such challenges further emphasize the importance of guidance from home care professionals, considering the caregivers’ ability to understand the information, their cultural beliefs, and the resources available for implementing care instructions.^34^

This study has some limitations, including issues related to the quality of retrospective data, such as incomplete records, omissions, erasures, and inappropriate terminology. Furthermore, as a cross-sectional study, causal relationships could not be established. However, strict inclusion and exclusion criteria were applied to medical records to mitigate these biases and used a validated instrument. Obtaining complete data can be challenging due to the reduced production of in-home care. Despite these limitations, this study significantly advances the understanding of home care and infection control.

## CONCLUSION

This study identified a predominance of male and elderly patients with a high incidence of neurological pathologies. Patients stayed longer in home care than in hospital settings, highlighting the importance of home treatment. The study found a high rate of HAIs at home, with respiratory infections being the most common. Clinical diagnosis was predominant, while the conduct of culture and antibiogram tests was limited. Patients assisted by female caregivers, with a tracheostomy, using invasive feeding devices for more than 6 months, and with a greater degree of dependence were more predisposed to infections. Furthermore, adult patients, those who received home care from young adult caregivers, those who extended home care durations, and those who required prolonged tracheostomy were at risk of contracting infections.

This study significantly enhances the understanding of home care and infection control, especially in an area where data are limited. The results provide valuable data on the profile of patients treated at home, the occurrence of HAIs at home, and the factors associated with these infections. It also highlights the need for preventive measures and specific interventions to improve the quality of home care and reduce the risk of infection.

Given the complexity of the home environment and the limitations identified in this study, future research should focus on identifying the risk factors of HAIs in home care settings. Further studies should explore more effective prevention strategies, refine diagnostic and treatment protocols, and assess the impact of specific interventions on infection rates. Furthermore, expanding the scope of the research to include a broader and more diverse patient sample and investigating home care practices in diverse regional contexts are crucial.
